# The Antioxidant and Enzyme Inhibitory Potential of *n*-Hexane-Extracted Oils Obtained from Three Egyptian Cultivars of the Golden Dewdrop *Duranta erecta* Linn. Supported by Their GC-MS Metabolome Analysis and Docking Studies

**DOI:** 10.3390/antiox11101937

**Published:** 2022-09-28

**Authors:** Shaimaa Fayez, Gokhan Zengin, Sara T. Al-Rashood, Mahmoud A. El Hassab, Wagdy M. Eldehna, Stefano Dall’Acqua, Omayma A. Eldahshan

**Affiliations:** 1 Department of Pharmacognosy, Faculty of Pharmacy, Ain Shams University, Cairo 11566, Egypt; 2 Department of Biology, Faculty of Science, Selcuk University, Konya 42130, Turkey; 3 Department of Pharmaceutical Chemistry, College of Pharmacy, King Saud University, Riyadh 11451, Saudi Arabia; 4 Department of Medicinal Chemistry, Faculty of Pharmacy, King Salman International University (KSIU), South Sinai 46612, Egypt; 5 Department of Pharmaceutical Chemistry, Faculty of Pharmacy, Kafrelsheikh University, Kafrelsheikh 33516, Egypt; 6 Department of Medicinal Chemistry, University of Padova, Via Marzolo 5, 35131 Padova, Italy; 7 Center for Drug Discovery Research and Development, Ain Shams University, Cairo 11566, Egypt

**Keywords:** *Duranta erecta*, GC-MS profiling, antioxidant activity, enzyme inhibitory effect, in-silico studies

## Abstract

*Duranta erecta* Linn. has a longstanding history for use in folk remedy for several disorders. Its hydroalcoholic extract has been investigated intensely in the treatment of many ailments, but to date very few data are presented to explain the pharmacological use of its oil. In this study, the chemical profiles of the leaf oils extracted from three Egyptian *Duranta erecta* cultivars, namely ‘Green’, ‘Golden edge’, and ‘Variegata’ are traced using GC-MS analysis. *D. erecta* ‘Green’ showed predominance of vitamin E (22.7%) and thunbergol (15%) whereas *D. erecta* ‘Golden edge’ and ‘Variegata’ contained tetratetracontane as a major component in their oils. The highest phenolic and flavonoid contents, displayed as gallic acid and rutin equivalents per gram oil, respectively, were observed in the ‘Golden edge’ and ‘Variegata’ cultivars, which was reflected by their strong DPPH and ABTS scavenging activities as well as the highest reducing power in both CUPRAC and FRAP assays. *D. erecta* ‘Green’ displayed better metal chelating potential, which may be attributed to its content of vitamin E. All cultivars showed similar enzyme inhibitory profiles. The best inhibition of α-glucosidase and α-amylase was observed by *D. erecta* ‘Green’. In silico studies of the major constituents docked on the active sites of the target enzymes NADPH oxidase, amylase, glucosidase, butyrylcholinesterase, and tyrosinase revealed high binding scores, which justified the biological activities of the tested oils.

## 1. Introduction

*Duranta erecta* (*D. repens*) Linn. (Verbenaceae) has long been traditionally used as a folk medicine in countries such as Brazil, Nigeria, India, Philippines, and Bangladesh [1]. The richness of the plant in phytoconstituents (ca. 64 isolated metabolite till now), in particular the iridoids, flavonoids, phenylethanoids, tannins, coumarinolignans, terpenoids, alkaloids, and sterols, make it of particular interest to natural products chemists. *D. erecta* hydroponics were of potential use for the removal of domestic pathogens from wastewater [2]. This flowering shrub (frequently named as golden dewdrop or sky flower or angel whisper or pigeon berry) is cultivated worldwide for its medicinal uses, which includes vermifuge [3], antiviral [4], antimicrobial [5], urolithiasis [6], antioxidant [5,7,8,9], anticancer [10,11], antimalarial [12], anticoagulant [13], antihyperglycemic [14,15,16], larvicidal [17], and antifungal [10,18] activities. However, most of the reported pharmacological effects were performed on the plant alcoholic extract rather than on its oil or *n*-hexane fraction [8,9,14].

Hyperglycemia induces a sequence of biochemical, phenotypic, and pathological alterations which could increase the risk of cardiovascular disorders and promotes the accumulation of free radicals and advanced glycation end-products leading to hyperglycemia-induced oxidative stress [19]. Insulin resistance and oxidative stress could exacerbate diabetic complications [20,21]. Several reports described the efficiency of *D. erecta* alcoholic extract and its pure isolated compounds on controlling glucose levels. Flavonoid-rich fractions displayed promising α-glucosidase and α-amylase inhibition, therefore reducing the sharp rise of glucose after meals [22,23]. The two isomers of 5′-methoxyisolariciresinol displayed different antioxidant and antiglycation behavior. Whereas the (+) isomer displayed potent antioxidant but mild (10%) antiglycation activity, the (−) isomer strongly inhibited the glucose-induced glycation of bovine serum albumin [24]. Another study [16] revealed that mice treated with *D. erecta* hydroalcoholic extract over thirty days showed significant improvement in their glucose tolerance, with a subsequent reduction in insulin resistance. Network pharmacology supported by in silico studies revealed that there are nearly 36 different metabolites in *D. erecta* that modulate up to 31 different targets and pathways involved in the pathogenesis of diabetes, with tyrosine phosphatase 1B and phosphatidylinositol 3-kinase-protein kinase B (PI_3_K-AKT) being the chiefly regulated target and pathway, respectively [25]. Patil et al. [15] showed that the hydroalcoholic extract of *D. erecta* enhanced the glucose uptake in yeast where in silico studies revealed that *α*-onocerin (one of the constituents of the extract) had the highest binding score to glucose transporter-2 protein. Isoprenylated flavonoids in *D. erecta* alcoholic extract significantly inhibited prolyl endopeptidase, whose levels seem to change in neuropsychiatric disorders such as Alzheimer, mania, and depression [26].

Since the majority of reports on *Duranta* presented the medical efficacy of its alcoholic extract especially as enzyme inhibitors, very few conclusive data were reported on the activity of its oil. Herein, we present the GC-MS metabolomic analysis of the oil obtained by the *n*-hexane extraction of the leaves of three different cultivars of *D. erecta* growing in Egypt, viz. ‘Green’, ‘Golden edge’, and ‘Variegata’. The total phenolic and flavonoid content of their extracts are studied with correlation to their in vitro antioxidant activities and their reducing power through DPPH, ABTS·+, CUPRAC, FRAP, metal chelation, and phosphomolybdenum assays. Moreover, their enzymatic inhibition properties are assessed on acetylcholine esterase, butyl cholinesterase, tyrosinase, amylase, and glucosidase enzymes. In silico studies are implemented which further supported the bioassays.

## 2. Materials and Methods

### 2.1. Extraction of the Essential Oils from D. erecta Cultivars by n-Hexane

The fresh leaves of three different cultivars of *Duranta erecta*, viz. ‘Green’, ‘Golden edge’, and ‘Variegata’ (ca. 100 g each) were collected in February 2022 from a botanical garden in Nasr City, Cairo. Authentication of the plant samples were kindly performed by Taxonomy Specialist Terase Labib, Consultant of Plant Taxonomy at the Ministry of Agriculture, and El-Orman Botanical Garden, Giza, Egypt. The leaves were dried, powdered, and macerated overnight in 100 mL *n*-hexane. The extract was filtered, and the filtrate was concentrated under vacuum. This process was repeated thrice over three consecutive days (3 × 100 mL) to yield 1.31 mg, 1.12 mg, and 1.09 mg of dark yellow (*D. erecta* ‘Green’), light yellow (*D. erecta* ‘Golden edge’), and light yellow (*D. erecta* ‘Variegata’) oily residues, respectively, which were subjected to GC-MS analysis and biological investigations. Voucher specimens (codes: PHG-P-DE-406, PHG-P-DE-407, and PHG-P-DE-408) were deposited at the Department of Pharmacognosy, Faculty of Pharmacy, Ain-Shams University, Cairo, Egypt.

### 2.2. GC-MS Analysis of the Extracted Oils

GC-MS analysis was performed on a Shimadzu QP2010 (Shimadzu Corporation, Kyoto, Japan) coupled to a quadrupole mass spectrometer. Separation was done on the low polarity diphenyl dimethyl polysiloxane Rtx-5MS (30 m × 0.25 mm i.d. × 0.25 µm thickness) capillary column (Restek, Bellefonte, PA, USA) in a split injection mode with a split ratio 1:15. Helium was the carrier gas flowing at a rate of 1.37 mL/min, sample injection volume 1 µL (diluted to 1% *v*/*v* in n-hexane), and oven and injector temperatures adjusted to 50 °C and 280 °C, respectively. Initial column temperature was set at 50 °C then gradually increased at a rate of 5 °C/min until reaching 300 °C, allowing metabolites of different boiling points and molecular weights to get separated at 70 eV EI ionization mode. Identification of oil components was based on comparison of their mass spectra and Kovat’s indices to those of a series of n-alkanes (C_8_–C_30_) injected under the same GC condition and to those reported in NIST online mass library, as well as matching with the literature [27,28,29,30].

### 2.3. Total Phenolic and Flavonoid Content

Folin-Ciocalteu and AlCl_3_ assays were used to determine the total phenolic and flavonoid contents, respectively [31]. For respective assays, results were expressed as gallic acid equivalents (mg GAEs/g dry extract) and rutin equivalents (mg REs/g dry extract).

### 2.4. Antioxidant Assays

Antioxidant assays were carried out according to previously reported methodologies [32,33]. The antioxidant potential was expressed as: mg Trolox equivalents (TE)/g extract in 2,2-diphenyl-1-picrylhydrazyl (DPPH) and 2,2′-azino-bis(3-ethylbenzothiazoline-6-sulfonic acid) (ABTS) radical scavenging, cupric reducing antioxidant capacity (CUPRAC), and ferric reducing antioxidant power (FRAP) tests, mmol TE/g extract in phosphomolybdenum assay (PBD), and mg ethylenediaminetetraacetic acid equivalents (EDTAE)/g extract in metal chelating assays (MCA).

### 2.5. Enzyme Inhibitory Assays

The enzyme inhibitory assays were carried out according to previously reported methodologies [32,33]. The acetylcholinesterase (AChE) and butyrylcholinesterase (BChE) inhibition was expressed as mg galanthamine equivalents (GALAE)/g extract; tyrosinase inhibition was expressed as mg kojic acid equivalents KAE/g extract; amylase and glucosidase inhibition were expressed as mmol acarbose equivalents (ACAE)/g extract.

### 2.6. Statistical Analysis

ANOVA (Tukey’s test) was used to determine if there were any differences in the tested extracts. The Pearson correlation test was used to examine the relationship between total bioactive components and biological activities. The statistical procedures were performed by using GraphPad 9.0.

### 2.7. In Silico Data

The X-ray 3D structures of NADPH oxidase, butyrylcholinesterase, tyrosinase, α-amylase, and α-glucosidase were downloaded from the protein data bank using the following IDs: 2cdu, 6esj, 5m8q, 4gqq and 3wy2, respectively. All the docking studies were conducted using MOE 2019^®^ software [34], which was likewise used to generate the 2D interaction diagrams between the docked ligands and their potential targets. The three identified major compounds were prepared using the default parameters and saved in a single mdb file. The active site of each target was determined from the binding of the corresponding co-crystalized ligand. The mdb file containing the three major compounds was finally docked into the active site of the five enzymes.

## 3. Results and Discussion

### 3.1. GC-MS Metabolomic Analysis of the Oils Obtained by the n-Hexane Extraction of Three Duranta Erecta Cultivars

A detailed chemoprofiling of the oils extracted by *n*-hexane from three Egyptian cultivars of *D. erecta* (*D. repens*), viz. ‘Green’, ‘Golden edge’, and ‘Variegata’ is presented in Table 1. GC-MS revealed the presence of some primary metabolites such as fatty acids (the majority of which in the form of esters), but many secondary constituents were detected including sterols, aliphatic long-chain alkanes and alkenes, vitamin E, and its isomers, as well as traces of phenolic components and ketonic derivatives. Mono- and sesquiterpene hydrocarbons were not detected in any of the hexane-extracted oils of the three cultivars. Previously, only two studies [35,36] reported on the GC-MS analysis of the hydrodistilled essential oil obtained from different organs of *D. repens,* which although obtained from the same country (Nigeria) and under the same extraction protocol (hydrodistillation), their metabolic profile was different.

**Figure 1:**
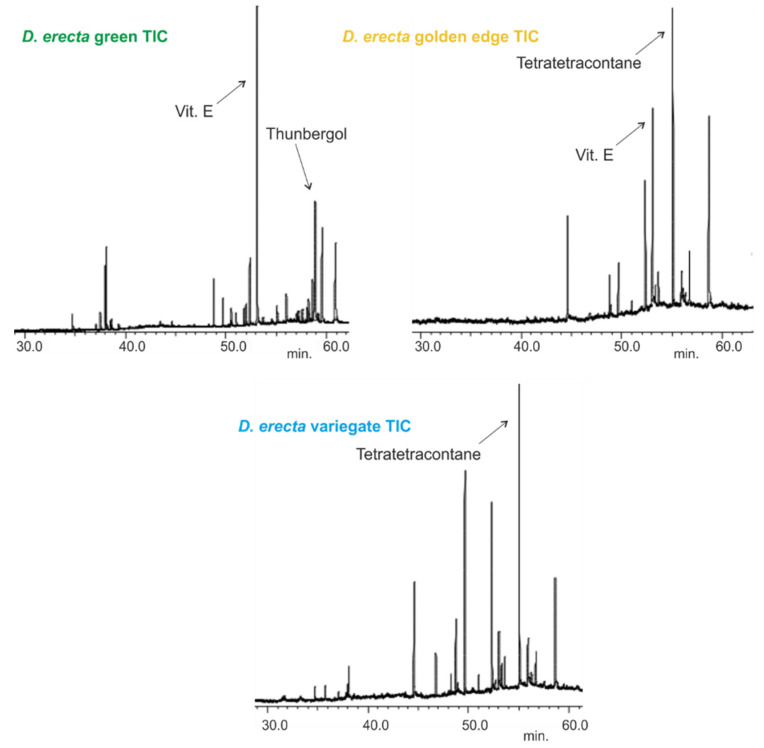
Total ion chromatograms (TIC) of the leaf oils extracted by *n*-hexane of three Egyptian *Duranta erecta* cultivars.

Thomas et al. [35] showed that limonene (11.6%), β-caryophyllene (7.5%), pentadecanal (6.7%), 1-octen-3-ol (5.1%), and α-humulene (5%) predominated the chromatogram of the oil obtained from the leaves whereas carvacrol (16.5%), β-caryophyllene (10.1%), 1,10-di-epicubenol (10.1%), and n-hexadecane (7%) were the major metabolites in the fruit oil. In contrast, Alade et al. [36] reported that the leaf oil was dominant in toluene (13.2%), 1-octen-3-ol (12.9%), and *p*-vinylanisole (11.7%), whereas styrene (52.5%) and palmitic acid (55.7%) were the major constituents of the oils obtained from the fruits and roots, respectively. The stem oil was in contrast rich in alkanes such as tetracosane (22.2%), tricosane (19.3%), pentacosane (18%), and docosane (14.2%). Here, the leaf oils of the three Egyptian *D. erecta* cultivars showed dissimilar metabolic profiles to a large extent (Figure 1).

**Figure 2:**
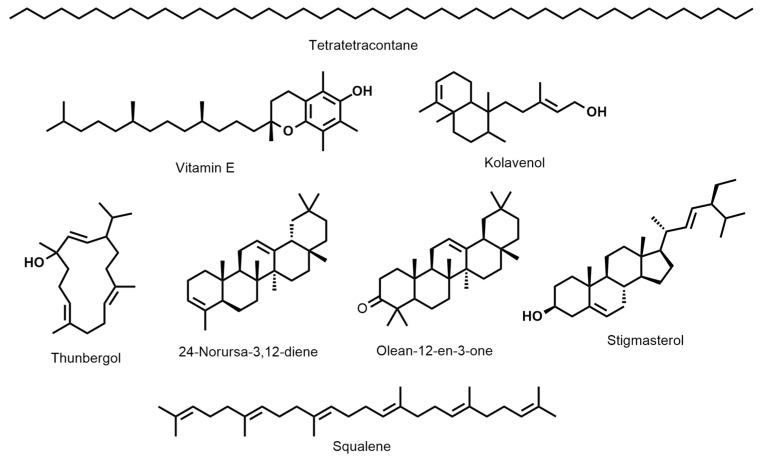
The structures of the major metabolites present in the hexane-extracted leaf oils in *D. erecta:* ‘Green’, ‘Golden egde’, and ‘Variegata’.

*D. erecta* ‘Green’ oil showed predominance in vitamin E and its isomers up to 22.7%, followed by the diterpene alcohol thunbergol (15%) and the triterpene steroidal alkene 24-norursa-3,12-diene (12.09%). Octadecatrienoic acid ethyl ester (5.4%) and olean-12-en-3-one (5.7%) were present in minor percentages. The leaf oil of *D. erecta* ‘Golden edge’ was predominantely rich in the unbranched long chain alkane, tetratetracontane, constituting ca. 61.97% of the whole oil followed by vitamin E, which represented 14.5%. Similarly, *D. erecta* ‘Variegata’ was highly rich in tetratetracontane (75.5%) but, in contrast to the ‘Golden edge’, stigmasterol (5.03%) and squalene (4.96%) were predominant in vitamin E (3.5%) in the oil of their leaves. The structures of the major metabolites are presented in Figure 2.

### 3.2. Assessment of the Total Phenolic and Flavonoid Content in Duranta-Derived Oils

Phenolic compounds play a key role in the management of many diseases [37,38,39,40]. The total phenolic and flavonoid content of the oils obtained from the three cultivars of *D. erecta* were determined by spectrophotometric methods. The results are presented in Table 2. The highest total phenolic and flavonoid content was found in *D. erecta* ‘Golden edge’, with 12.07 mg GAE/g and 8.51 mg RE/g, respectively. *D. erecta* ‘Green’ contained the lowest level of these bioactive compounds, which were ca. 8.51 mg GAE/g and 1.49 mg RE/g, respectively. The content of the total phenolic compounds was clearly dependent on the tested *Duranta* cultivars. Consistent with our results, the concentration of flavonoids and phenolics were different in the cultivars of a plant species.

To the best of our knowledge, there is no scientific data on *Duranta* oils, although the content of the total bioactive compounds in some of its members have been reported in several publications [5,8,41]. Recently, some concerns were raised about the spectrophotometric assays, where some compounds do not react with the reagents used [42,43]. Therefore, the results obtained from spectrophotometric assays need to be further confirmed by chromatographic and spectroscopic techniques such as HPLC and NMR, respectively.

### 3.3. In Vitro Antioxidant Assays

Currently, natural antioxidants are replaced with synthetic alternatives, which appeared to have adverse effects on humans [43]. In this regard, we demonstrated the antioxidant potential of the oils of the three *D. erecta* cultivars and the results are presented in Table 2 and Table 3. Different antioxidant assays are required to get a complete picture of the antioxidant potential of the tested samples. Therefore, six complementary assays (DPPH, ABTS, CUPRAC, FRAP, MCA, and PBD) were performed to assess the antioxidant properties of *Duranta* oils. The free radical scavenging potential was assessed using DPPH and ABTS. *D. erecta* ‘Golden edge’ showed the strongest DPPH scavenging ability (12.93 mg TE/g), whereas *D. erecta* ‘Variegata’ displayed the best ABTS scavenging capacity (8.98 mg TE/g). The results of DPPH and ABTS revealed that *D. erecta* ‘Green’ had no ability to scavenge free radicals. The reducing power of the oils, which is their ability to donate an electron to stabilize free radicals, were studied using CUPRAC (ability to reduce Cu^2+^ to Cu^+^) and FRAP (ability to reduce Fe^3+^ to Fe^2+^) assays. The best reducing power was observed for *D. erecta* ‘Variegata’ (CUPRAC: 36.40 mg TE/g; FRAP: 21.07 mg TE/g), followed by ‘Golden edge’ and ‘Green’.

**Figure 3:**
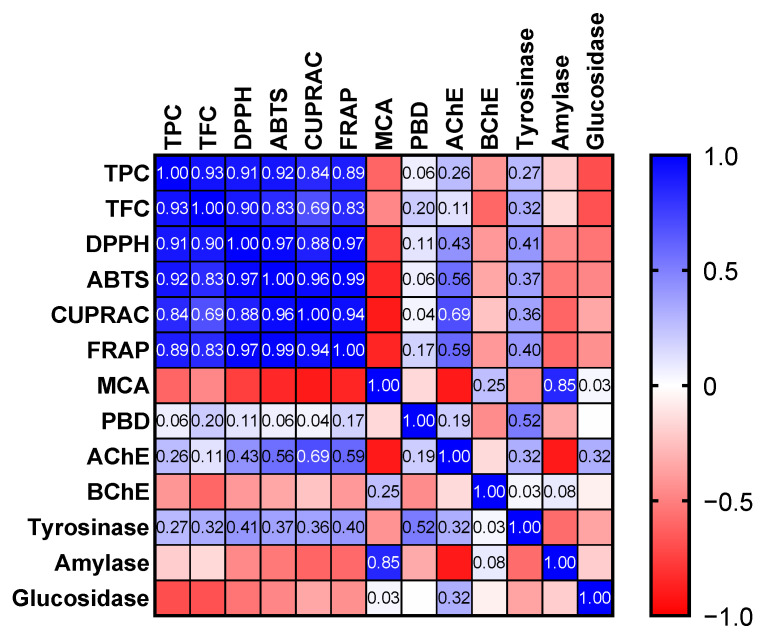
Pearson correlation values between biological activity assays (*p* < 0.05). TPC: Total phenolic content; TFC: Total flavonoid content; ABTS: 2,2′-azino-bis(3-ethylbenzothiazoline-6-sulphonic acid; DPPH: 1,1-diphenyl-2-picrylhydrazyl; CUPRAC: Cupric reducing antioxidant capacity; FRAP: Ferric reducing antioxidant power; MCA: Metal chelating ability; PBD: Phosphomolybdenum; AChE: acetylcholinesterase; BChE: butyrylcholinesterase.

Apparently, the free radical scavenging and reducing abilities of the tested oils are consistent with their levels of total bioactive compounds. This fact was also confirmed by Pearson’s correlation analysis, and strong correlation values (R > 0.7) were determined (Figure 3). Consistent with our results, good correlation values between these parameters have been reported by several researchers [44,45,46]. The phoshomolybdenum assay involves the conversion of Mo (VI) to Mo (V) by antioxidants in the acidic condition. Thus, the assay is known as one of reducing power assays. In addition, since all antioxidant compounds could be active, the assay is known as the total antioxidant assay [43]. As can be seen in Table 3, the tested samples exhibited very similar effects in the assay (*p* > 0.05).

The chelation of transition metals is an important mechanism and prevents the formation of the most dangerous hydroxyl radical. The best ability for metal chelation was observed in *D. erecta* ‘Green’ with 16.37 mg EDTAE/g, followed by ‘Golden edge’ (10.71 mg EDTAE/g) and ‘Variegata’ (2.77 mg EDTAE/g). The observed metal chelating ability could be explained by the presence of tocopherol in the tested oil. These findings are in accordance with those in the literature where tocopherol has been reported as a good metal chelator [47,48,49]. Although few previous works [36] have explained the significant antioxidant properties of the methanol extracts of some members of *Duranta*, the antioxidant activities of the oils of the three cultivars of *D. erecta* are moderate to some extent.

### 3.4. Enzyme Inhibitory Assay

Some diseases, such as Alzheimer’s disease, diabetes mellitus, or obesity, affect millions of people, and their prevalence is increasing every day. With this in mind, new and effective strategies are becoming increasingly popular as a subject of study. Enzymes are critical in managing the diseases mentioned above. The symptoms of the diseases could be controlled by inhibiting important enzymes. Some enzymes, such as acetylcholinesterase, amylase, and lipase, have been identified as targets for certain diseases, such as Alzheimer’s, diabetes, and obesity, respectively. In this regard, several synthetic enzyme inhibitors have been approved in the treatment of diseases, but the majority of which have unpleasant side effects [50,51,52,53]. Therefore, natural sources could be considered as treasures that need detailed exploration. We tested the enzyme inhibitory properties of the oils obtained from the three *Duranta erecta* cultivars against cholinesterases (AChE and BChE), tyrosinase, amylase, and glucosidase. The results are presented in Table 4. In general, the tested oils exhibited similar inhibitory activities against the enzymes. Although BChE was inhibited by all the tested oils (6.41–6.65 mg GALAE/g), AChE was only inhibited by *D. erecta* ‘Variegata’. Tyrosinase is the main catalyst in the synthesis of melanin and its inhibition is important to treat hyperpigmentation problems. As presented in Table 4, the tested oils exhibited, to large extent, similar anti-tyrosinase effects (56.92–61.96 mg KAE/g, *p* > 0.05).

Amylase and glucosidase were selected as targets for antidiabetic effects and the best amylase inhibition was recorded by *D. erecta* ‘Green’ and ‘Golden edge’ with 0.32 mmol ACAE/g. However, the strongest glucosidase inhibition was observed in *D. erecta* ‘Green’ (1.31 mmol ACAE) and ‘Variegata’ (1.24 mmol ACAE). It is clear from Figure 3 that the total content of phenols and flavonoids weakly correlate or even did not correlate with the abilities to inhibit enzymes. Consistent with our findings, several researchers also reported a weak correlation between total bioactive compounds and enzyme inhibition effects [54,55,56]. In the literature, available data on the enzyme inhibitory properties of the members of the genus *Duranta* is limited [22,23,56] and no previous study has focused on its oils. Thus, the presented results could be a scientific basis for further applications with *D. erecta* oils.

### 3.5. Docking Results

The three major compounds, viz. α-tocopherol, thunbergol, and tetratetracontane were docked into the active site vicinity of the five enzymes (i.e., NADPH oxidase, butyrylcholinesterase, tyrosinase, α-amylase, and α-glucosidase). As presented in Table 5, all compounds achieved acceptable binding scores when docked with the five targets.

**Figure 4:**
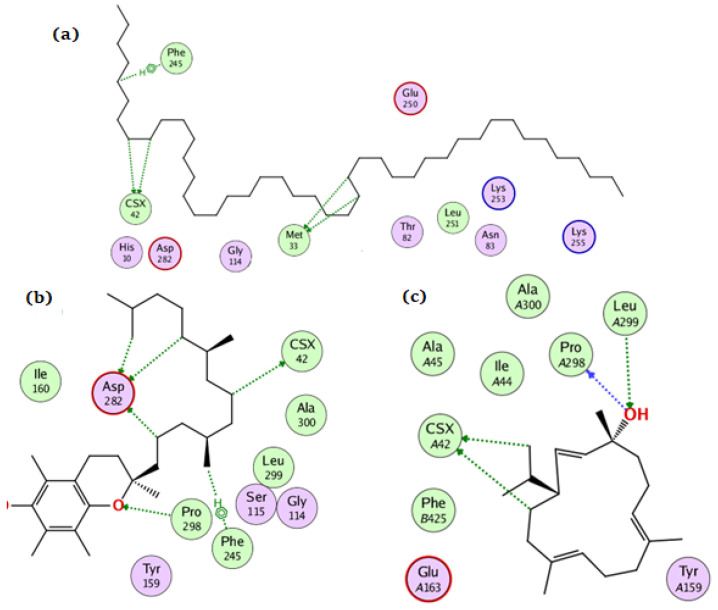
The docking of (**a**) tetratetracontane, (**b**) α-tocopherol, and (**c**) thunbergol on the active site of NADPH oxidase enzyme (PDB code: 2cdu).

For the NADPH oxidase, the compounds tetratetracontane, α-tocopherol, and thunbergol achieved docking scores of −10.3, −13.9, and −11.4 kcal/mol, respectively. Tetratetracontane interacted with the amino acid residues Cys42, Phe245, and Met33, whereas α-tocopherol interacted with Cys42, Pro298, Phe245, and Asp282 (Figure 4). Thunbergol bound to NADPH oxidase through hydrogen bond interactions with Cys42, Pro298, and Leu299.

**Figure 5:**
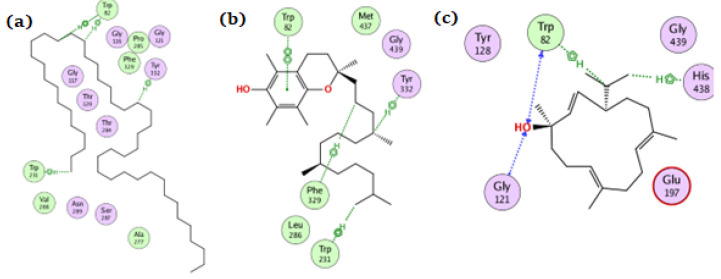
The docking of (**a**) tetratetracontane, (**b**) α-tocopherol, and (**c**) thunbergol on the active site of BChE enzyme (PDB code: 6esj).

For the BChE enzyme, tetratetracontane, α-tocopherol, and thunbergol achieved docking scores of −11.1, −13.9, and −9.4 kcal/mol, respectively. As depicted from Figure 5, tetratetracontane interacted with BChE at Trp82, Tyr332, and Trp231, whereas α-tocopherol showed interactions at Trp82, Tyr332, Phe329, and Trp231. On the other hand, thunbergol interacted with BChE at Trp82, Gly121, and His438 residues.

**Figure 6:**
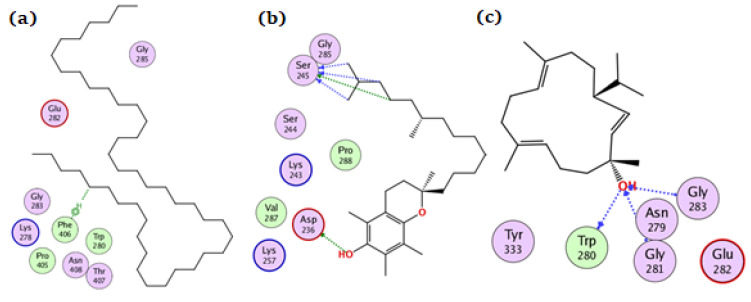
The docking of (**a**) tetratetracontane, (**b**) α-tocopherol, and (**c**) thunbergol on the active site of amylase enzyme (PDB code: 4gqq).

Tetratetracontane, α-tocopherol, and thunbergol achieved docking scores of −7, −7.8, and −7.2 kcal/mol, respectively, for the amylase enzyme. Tetratetracontane interacted with amylase only through Phe406, α-tocopherol interacted at Asp236, Ser245, and Gly285 while thunbergol bounded at Trp280, Gly281, and Gly283 (Figure 6).

**Figure 7:**
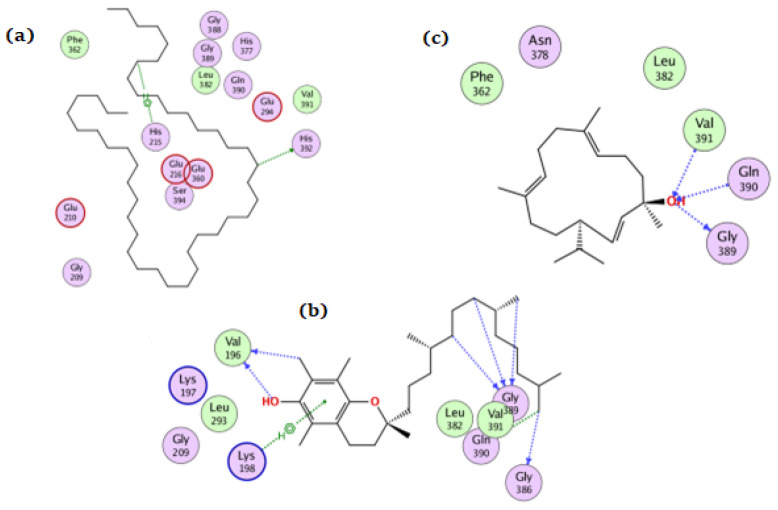
The docking of (**a**) tetratetracontane, (**b**) α-tocopherol, and (**c**) thunbergol on the active site of tyrosinase enzyme (PDB code: 5m8q).

For tyrosinase, tetratetracontane, α-tocopherol, and thunbergol achieved docking scores of −8.5, −10.3, and −8.7 kcal/mol, respectively. Figure 7 showed the interaction of the three compounds with tyrosinase in which tetratetracontane interacted at His215 and His392 residues, α-tocopherol interacted at Val196, Lys198, Gly386, Gln390, and Gly389 residues, whereas thunbergol interacted at Val391, Gln390, and Gly389.

**Figure 8:**
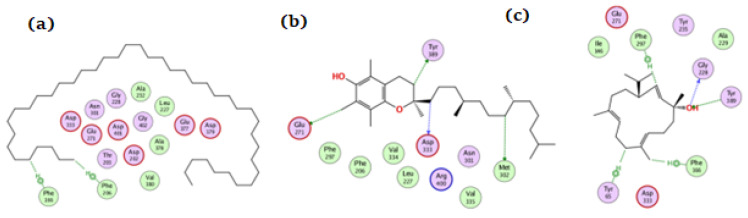
The docking of (**a**) tetratetracontane, (**b**) α-tocopherol, and (**c**) thunbergol on the active site of glucosidase enzyme (PDB code: 3wy2).

On glucosidase, tetratetracontane, α-tocopherol, and thunbergol achieved docking scores of −12.1, −13.5, and −13.3 kcal/mol, respectively. Figure 8 showed the interaction of the compounds with glucosidase where tetratetracontane bounded at the amino acid residues Phe166 and Phe206, α-tocopherol bounded at Glu271, Met302, Tyr389, and Asp333, whereas thunbergol interacted at Phe166, Gly228, Tyr65, Tyr389, and Phe297 residues. The docking data justified the biological results where a synergetic effect for all the components of the extract could be seen.

## 4. Conclusions

*Duranta erecta* is an important herb with several medicinal and industrial applications. Phytochemical and pharmacological studies have previously reported on its hydroalcoholic extract; however, few documented its oil and properties. GC-MS investigations on three different cultivars of *D. erecta* growing in Egypt showed significant variations in their constituent profiles. Mono- and sesquiterpene hydrocarbons were almost absent in *Duranta* oil with predominance in fatty esters, acids, alkanes, alkenes, sterols, alcohols, tocopherols, and some phenolics. In vitro antioxidant assays showed that the oils displayed good radical scavenging activities and promising reducing power, which was likewise supported by the in silico data. Therefore, we strongly recommend further investigations on *Duranta* oils, such as toxicity studies, animal testing, and bioavailability.

## Figures and Tables

**Table 1:** GC-MS profiling of the hexane extracts of three different cultivars of *Duranta erecta,* viz. ‘Green’, ‘Golden edge’, and ‘Variegata’ on RTX-5 capillary column showing the relative percentage of metabolites.

Peak No.	R_t_ (min.)	KI *	Identification		Peak Area (%)	
		Cyclic/Steroidal/Fatty Acid Ester		*D. erecta* ‘Green’	*D. erecta* ‘Golden Edge’	*D. erecta* ‘Variegata’
1	34.7	1994	Palmitic acid, ethyl ester	0.89	-------	-------
2	38.0	2171	9,12,15-Octadecatrienoic acid, ethyl ester, (*Z*,*Z*,*Z*)	5.41	-------	2.06
3	38.5	2193	Isopropyl linoleate	0.67	-------	-------
4	38.6	2200	9,12,15-Octadecatrienoic acid, ethyl ester, (*Z*,*Z*,*Z*)	0.67	-------	-------
5	43.4	2480	Glycidyl palmitoleate	0.21	-------	-------
6	44.6	2554	Di-*n*-octyl phthalate	0.32	8.09	8.29
7	58.0	3555	Lup-20(29)-en-3-ol, acetate, (3*β*)	1.31	-------	-------
8	59.1	3627	(−)-Isolongifolol, acetate	0.78	-------	-------
9	60.8	3749	Kolavenol acetate	9.0	-------	-------
		Total esters		19.26	8.09	10.35
		**Cyclic/Steroidal (sterols)/Fatty alcohols**				
10	37.0	2115	Phytol	0.29	-------	-------
11	37.5	2139	2-(*Z*,*Z*)-9,12-octadecadienyloxyethanol	0.98	-------	-------
12	39.3	2239	2-Octylcyclopropene-1-heptanol	0.23	-------	-------
13	50.4	2954	*trans*-Geranylgeraniol	0.24	-------	-------
14	55.0	3294	Stigmasterol	2.01	-------	-------
15	55.9	3352	*β*-Sitosterol	2.54	-------	-------
16	55.9	3347	Ergosta-7,22-dien-3-ol, (3*β*.,22*E*)-	-------	3.50	-------
17	55.9	3346	Stigmasterol	-------	-------	5.03
18	57.0	3446	(−)-Globulol	0.82	-------	-------
19	57.2	3481	3,7,11,15-Tetramethyl hexadeca-1,6,10,14-tetraen-3-ol	1.22	-------	-------
20	58.2	3564	Humulane-1,6-dien-3-ol	2.52	-------	-------
21	58.8	3610	Thunbergol	15.35	-------	-------
		Total alcohols		26.2	3.50	5.03
		**Fatty acids**				
22	37.9	2164	9,12-Octadecadienoic acid (*Z*,*Z*)-	3.62	-------	-------
			Total acids	3.62	-------	-------
		**Aliphatic/Cyclic/Steroidal alkanes**				
23	46.7	2695	*n*-Tetratetracontane	-------	-------	2.89
24	48.2	2795	*n*-Tetratetracontane	-------	-------	1.20
25	49.6	2897	*n*-Tetratetracontane	1.60	3.70	14.6
26	51.0	2997	*n*-Tetratetracontane	0.71	-------	1.22
27	52.3	3098	*n*-Tetratetracontane	3.70	9.17	12.4
28	53.2	3168	Hentriacontane, 3-methyl	-------	-------	1.42
29	53.3	3170	Tritetracontane	-------	1.38	-------
30	53.6	3198	2-methyloctacosane	0.39	2.52	-------
31	53.6	3194	*n*-Tetratetracontane	-------	-------	2.16
32	55.0	3296	*n*-Tetratetracontane	-------	26.4	25.2
33	56.6	3394	*n*-Tetratetracontane	-------	-------	3.06
34	56.7	3395	2-methyloctacosane	-------	5.07	-------
35	58.6	3595	*n*-Tetratetracontane	-------	22.7	11.8
			Total alkanes	6.4	70.94	75.95
		**Aliphatic/Cyclic/Steroidal alkenes**				
36	48.7	2834	Squalene	2.66	2.90	4.96
37	57.5	3520	24-Norursa-3,12-diene	1.36	-------	-------
38	59.5	3657	24-Norursa-3,12-diene	10.73	-------	-------
			Total alkenes	14.75	2.90	4.96
		**Vitamins**				
39	50.5	2960	*δ*-Tocopherol	1.03	-------	-------
40	51.9	3069	γ-Tocopherol	1.35	-------	-------
41	53.1	3154	Vitamin E	20.32	14.5	3.51
			Total vitamins	22.7	14.5	3.51
		**Phenolics**				
42	51.7	3054	(*R*)-6-Methoxy-2,8-dimethyl-2-((4*R*,8*R*)-4,8,12-trimethyltridecyl) chroman	1.05	-------	-------
			Total phenolics	1.05	-------	-------
		**Cyclic/Steroidal ketones**				
43	58.6	3591	Olean-12-en-3-one	5.71	-------	-------
			Total ketones	5.71	-------	-------
		**Unknown**				
44	58.4	3578	Unknown	0.29	-------	-------
Total identified				99.63	100.0	100.0

* KI (Kovat’s index) was identified with respect to a series of *n*-alkanes (C_8_–C_30_) on the RTX-5 capillary column. R_t_ stands for the retention time. Text written in bold are those phytochemical classes identified in the *n*-hexane extracts of *Duranta erecta* cultivars.

**Table 2:** Total phenolic (TPC), flavonoid (TFC), and radical scavenging (DPPH and ABTS) abilities of the tested oils.

Samples	TPC (mg GAE/g)	TFC (mg RE/g)	DPPH (mg TE/g)	ABTS (mg TE/g)
*D. erecta* ‘Green’	8.51 ± 0.16 ^b^	1.49 ± 0.10 ^c^	Na	Na
*D. erecta* ‘Golden edge’	12.07 ± 0.80 ^a^	2.28 ± 0.15 ^a^	12.93 ± 1.10 ^a^	8.19 ± 0.76 ^a^
*D. erecta* ‘Variegata’	11.18 ± 0.60 ^a^	1.97 ± 0.11 ^b^	11.76 ± 0.44 ^a^	8.98 ± 0.64 ^a^

Values are reported as mean ± SD. of three parallel measurements. GAE: Gallic acid equivalent; RE: Rutin equivalent; TE: Trolox equivalents; TPC: Total phenolic content; TFC: Total flavonoid content; na: not active. Different superscripts indicate significant differences in the tested samples (*p* < 0.05). DPPH: 2,2-Diphenyl-1-picrylhydrazyl; ABTS: 2,2′-azino-bis(3-ethylbenzothiazoline-6-sulfonic acid.

**Table 3:** Reducing abilities, metal chelating, and total antioxidant ability (by phosphomolybdenum assay) of the tested oils.

Samples	CUPRAC (mg TE/g)	FRAP (mg TE/g)	MCA (mg EDTAE/g)	PBD (mmol TE/g)
*D. erecta* ‘Green’	27.48 ± 0.29 ^b^	13.86 ± 0.66 ^b^	16.37 ± 1.11 ^a^	0.80 ± 0.08 ^a^
*D. erecta* ‘Golden edge’	33.80 ± 2.14 ^a^	20.21 ± 0.28 ^a^	10.71 ± 0.53 ^b^	0.80 ± 0.05 ^a^
*D. erecta* ‘Variegata’	36.40 ± 1.05 ^a^	21.07 ± 0.03 ^a^	2.77 ± 0.14 ^c^	0.82 ± 0.05 ^a^

Values are reported as mean ± SD. Of three parallel measurements. TE: Trolox equivalents; EDTAE: EDTA equivalent. Different superscripts (a–c) indicate significant differences in the tested samples (*p* < 0.05). CUPRAC: Cupric reducing antioxidant capacity; FRAP: The ferric reducing antioxidant power; MCA: Metal chelation assay; PBD: Phosphomolybdenum assay.

**Table 4:** Enzyme inhibitory properties of the tested oils.

Samples	AchE (mg GALAE/g)	BChE (mg GALAE/g)	Tyrosinase (mg KAE/g)	Amylase (mmol ACAE/g)	Glucosidase (mmol ACAE/g)
*D. erecta* ‘Green’	Na	6.65 ± 0.15 ^a^	56.92 ± 3.35 ^a^	0.32 ± 0.01 ^a^	1.31 ± 0.01 ^a^
*D. erecta* ‘Golden edge’	Na	6.41 ± 0.40 ^a^	60.54 ± 2.36 ^a^	0.32 ± 0.01 ^a^	1.24 ± 0.03 ^b^
*D. erecta* ‘Variegata’	2.31 ± 0.03	6.45 ± 0.26 ^a^	61.96 ± 8.52 ^a^	0.28 ± 0.02 ^b^	1.30 ± 0.02 ^a^

Values are reported as mean ± SD of three parallel measurements. GALAE: Galatamine equivalent; KAE: Kojic acid equivalent; ACAE: Acarbose equivalent; Na: not active. Different superscripts (a,b) indicate significant differences in the tested samples (*p* < 0.05). AChE: acetylcholinesterase; BChE: butyrylcholinesterase.

**Table 5:** The docking scores of tetratetracontane, α-tocopherol, and thunbergol on the active sites of the enzymes BChE, amylase, NADPH oxidase, tyrosinase, and glucosidase.

Compound	Docking Scores (kcal/mol)				
	NADPH ox (2cdu)	BChE (6esj)	Amylase (4gqq)	Tyrosinase (5m8q)	Glucosidase (3wy2)
Tetratetracontane	−10.3	−11.1	−7.0	−8.5	−12.1
*α*-Tocopherol	−13.9	−13.9	−7.8	−10.3	−13.5
Thunbergol	−11.4	−9.4	−7.2	−8.7	−13.3

## Data Availability

The data presented in this study are available on request from the corresponding author.
